# Supplementary material for: A Global Screen for Assembly State Changes of the Mitotic Proteome by SEC-SWATH-MS
Source: Cell Syst. 2020 Feb 26;10(2):133–155.e6. doi: 10.1016/j.cels.2020.01.001 (PMC7042714; doi:10.1016/j.cels.2020.01.001)

Q86Y46 | K2C73_HUMAN | KRT73 K6IRS3 KB36 KRT6IRS3
Monomer MW [kDa]: 58.923 Monomer expected elution fraction: 45

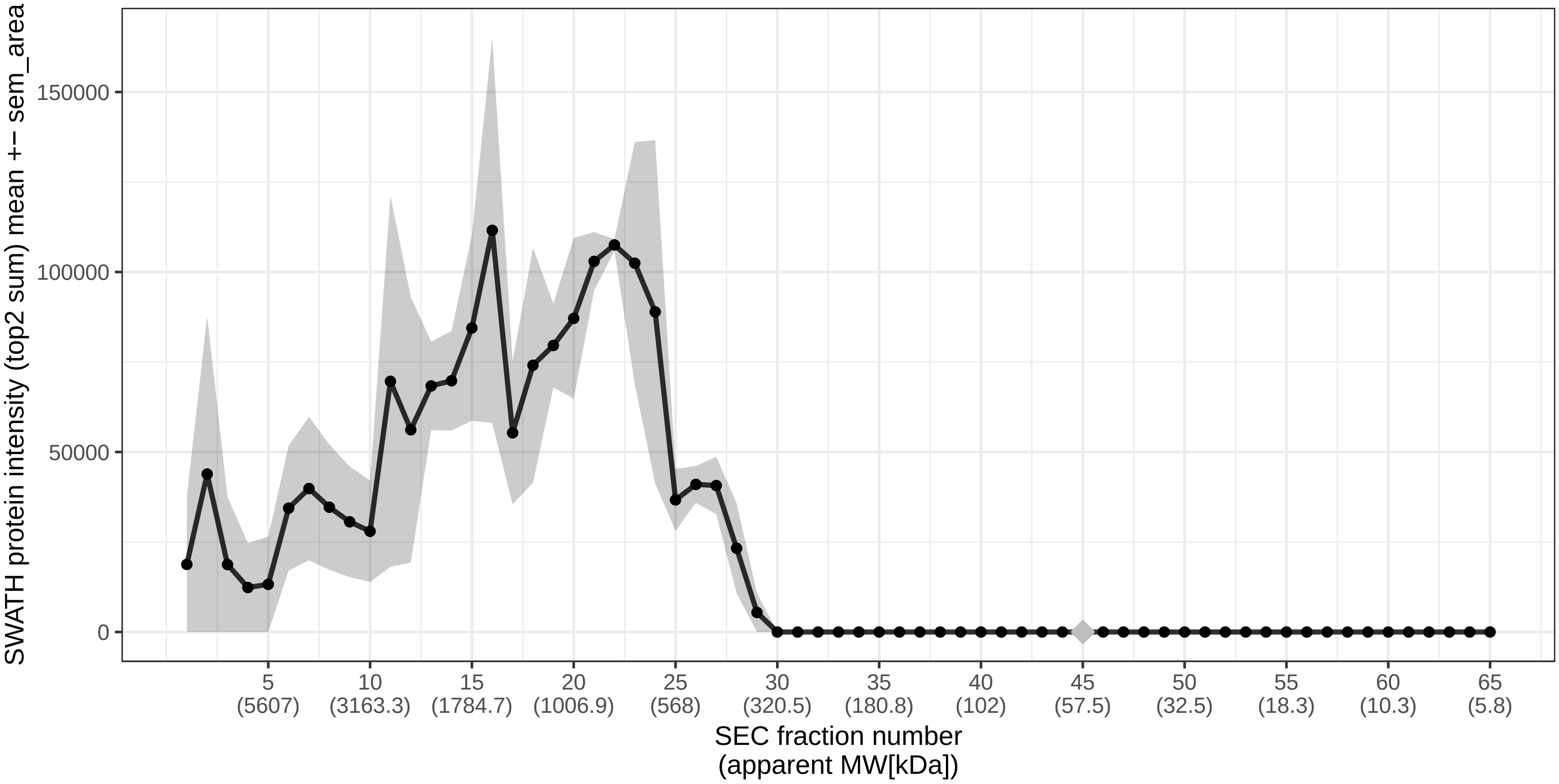

Supplement: Data S1. SEC-SWATH-MS Protein Chromatograms, Related to Figure 1 [file mmc6.zip › SECchrom_Q86Y46_K2C73_HUMAN_KRT73_K6IRS3_KB36_KRT6IRS3.pdf]
